# The Impact of COVID-19 on the Financial Performance of Largest Teaching Hospitals

**DOI:** 10.3390/healthcare11141996

**Published:** 2023-07-11

**Authors:** Karima Lalani, Jeffrey Helton, Francine R. Vega, Marylou Cardenas-Turanzas, Tiffany Champagne-Langabeer, James R. Langabeer

**Affiliations:** 1 Center for Health Systems Analytics, D. Bradley McWilliams School of Biomedical Informatics, The University of Texas Health Science Center at Houston (UTHealth Houston), Houston, TX 77030, USA; francine.r.vega@uth.tmc.edu (F.R.V.); maria.cardenasturanzas@uth.tmc.edu (M.C.-T.); tiffany.champagne@uth.tmc.edu (T.C.-L.); james.r.langabeer@uth.tmc.edu (J.R.L.); 2 Department of Health Administration, University of Colorado at Denver Business School, Denver, CO 80202, USA; jeffrey.helton@ucdenver.edu

**Keywords:** COVID-19, teaching hospital, financial performance

## Abstract

The COVID-19 pandemic disrupted hospital operations. Anecdotal evidence suggests financial performance likewise suffered, yet little empirical research supports this claim. This study aimed to explore the impact of the pandemic on the financial performance of the most prominent academic hospitals in the United States. Data from the 115 largest major teaching hospitals in the United States were extracted from the American Hospital Directory for three years (2019–2021). We hypothesized that the year and region would moderate the relationship between a hospital’s return on assets (financial performance) and specific operational variables. We found evidence through descriptive statistics and multivariate moderated regressions that financial positions rebounded in 2021, mainly through reductions in adjusted full-time employees and liabilities and an increase in non-operating income. Our results also found that the Midwest region significantly outperformed the other three regions, particularly in terms of lower salaries and operational expenses. These findings suggest potential for future initiatives encouraging efficiency and finding alternate sources of income beyond patient income. Hospitals should focus on improving financial reserves, building out non-operational revenue streams, and implementing operational efficiencies to foster better financial resiliency. These suggestions may enable healthcare administrators and facilities to adapt to future pandemics and environmental turbulence.

## 1. Introduction

### 1.1. Background

Teaching hospitals in the United States are under continuous financial pressure, largely due to a more complex case mix, declining reimbursement for patient care services, and increasing expenses associated with staffing and graduate medical education [1]. Uncertainty in the hospital’s external environment, such as geographic location and market concentration, has also been shown to negatively impact short- and long-term financial performance [2]. The swift progression of the COVID-19 pandemic has been credited with creating unprecedented economic challenges for hospitals, namely abrupt reductions in higher-revenue elective and outpatient services, an increase in the cost of supplies and labor, as well as the influx of patients admitted into intensive care units [3,4,5,6,7,8].

### 1.2. Literature Review

The healthcare system in the United States is best described as a hybrid since it combines public (in the form of Medicare and Medicaid) and private (in the form of health insurance plans) funding sources. Most healthcare in the United States is financed and delivered through patients’ own contributions or through the private insurance market [9]. In 2019, roughly half of Americans had private insurance through their employers (group insurance), 6% had private insurance through health insurance marketplaces (nongroup insurance), 20% had Medicaid coverage, 14% had Medicare coverage, and 1% had other public insurance (e.g., Veterans Health Administration [VHA] and Military Health Service [MHS]) [10]. U.S. teaching hospitals are unique establishments considered the cornerstone of the American healthcare system. Teaching hospitals deliver a critical combination of graduate medical education (GME), clinical research, and inpatient and ambulatory services [1,2,11]. Because of their relationship with major academic medical centers, teaching hospitals have considerable donor capital available [4]. This capital, however, tends to be restricted for a specific use, limiting the ability of teaching hospitals to use these funds to offset general operating losses. There are roughly 113,995 residents and 4650 trainees in ACGME and AOA accredited programs instructed at U.S. teaching hospitals, with Medicare compensating 89,578 at or below the cap payment amount specified in 1997 [12].

Breaking down the financial performance of teaching hospitals over the past thirty years exposes consistent financial distress within these organizations. Decade-long financial challenges include the implementation of the prospective payment system in the 1980s, reductions in Medicare payment rates in the 1990s, and caps on graduate medical education (GME) payments created by the Balanced Budget Act of 1997 [13]. In addition to regulatory changes, teaching hospitals also faced reduced revenue due to bad debts, uninsured patients, and managed care plan payment authorization and coverage requirements. While these impacts are not wholly unique to teaching hospitals, the magnitude of those impacts is greater in teaching hospitals, which see larger proportions of uninsured and underinsured patients [14] and are often situated in economically depressed neighborhoods of major cities [13]. However, empirical research has shown that hospitals serving remote rural areas are more likely to face financial difficulties [4].

These pressures were compounded by challenges in lending conditions and competition from large multi-hospital systems that do not support graduate medical education [3,6,15]. The pandemic may have exacerbated these financial challenges, as many patients avoided receiving preventive or elective healthcare services due to concerns about disease transmission and local ordinances, resulting in the loss of higher-paying elective patient volumes and revenues [3,16]. These factors, combined with state or federal guidelines to shut down non-essential service lines, translated into decreased revenue for many healthcare delivery organizations.

Concerned stakeholders worry about sustainability for teaching hospitals that generally operate as non-profit and public entities. These types of hospital facilities saw a change in their expected financial outlook, being downgraded from stable to negative in March 2020 [3,6,16]. Additionally, some hospitals cared for a disproportionate number of patients with COVID-19, while others cared for a greater number of low-income and uninsured patients. Teaching hospitals saw a combination of all these events [3,7,17].

COVID-19 care prompted higher operating expenses for necessary supplies and rapidly escalating labor costs [5,6]. Overall, hospitals in the United States experienced a total loss of over $200 billion because of an estimated 45% decrease in operating revenue [18] between 1 March and 30 June, 2020. We intend to further explore the financial performance of teaching hospitals during the pandemic era with an eye toward measuring the extent of such predicted impacts on this subset of U.S. hospitals by comparing the year before the pandemic (2019) with the first two years of the pandemic era (2020 and 2021).

According to data from 2018, the operating margin for hospitals averaged 2.0%, with a median asset-to-liability ratio of 2.1, indicating a nominal ability to cover liabilities [4]. The median number of days of cash on hand (indicative of a hospital’s ability to pay overtime) was 52.4 days. Teaching hospitals that had relatively higher operating cash flow margins and greater days of cash on hand were anticipated to outperform other teaching hospitals that were in more perilous situations throughout the COVID-19 pandemic [3,4,6].

For the last few decades, around a third of hospitals throughout the country have had negative operating margins [19]. Among the largest hospitals, profitability tends to be positive only when considering non-operating revenue contributions [20]. During COVID-19, the severity of the disease and lack of outpatient volumes created worsening performance [21].

Since teaching hospitals deliver a tripartite role (GME, research and clinical services) and provide more than 7.1 million jobs nationwide, understanding the impact of COVID-19 on this part of the healthcare system and their recovery is significant for public health [2]. In this study, our objective was to analyze the impact of COVID-19 on the financial performance of the largest teaching hospitals in the United States by comparing whether key measures of financial performance have improved during the most recent fiscal year, post-COVID-19.

## 2. Materials and Methods

### 2.1. Study Design, Study Sample

This study was a retrospective, cross-sectional observational study using publicly available secondary data from 2019 to 2021. We focused exclusively on major teaching hospitals, defined by the American Medical Colleges’ Council on Teaching Hospitals and Health Systems (COTH) as facilities with four or more medical school residency programs that are affiliated with accredited U.S. medical schools [22]. The list of hospitals was pulled from the American Hospital Directory dataset to ensure affiliation as a teaching hospital [23]. Upon extracting the data, the hospitals were ranked according to gross and net revenues in the most recent fiscal year. We excluded Veterans Administration (VA) hospitals and specialty and children’s hospitals since these facilities are not subject to the same Prospective Payment System (“PPS”) reimbursement mechanism as the general acute care teaching hospitals included in this study.

Targeting a sample size of approximately 100 hospitals, we extracted data for the 130 largest short-term acute care teaching hospitals, with the assumption of incomplete data in some records. We eliminated those with missing or incomplete data, which resulted in a final sample of 104 hospitals. We extracted key control, financial and operating data from the Centers for Medicare and Medicaid Services (CMS) Healthcare Cost Report Information System, or “HCRIS”, annual cost report dataset for the three years 2019–2021 [24,25,26]. As this is publicly available data, this study was exempt from the Institutional Review Board’s human subject protection review.

### 2.2. Study Variables

Fiscal year 2021 is the most recent complete year of available data from this source at the time of this writing. All variables were adjusted to account for hospital size and volume, primarily based on adjusted patient days, a measure that considers inpatient and outpatient service volumes in a hospital [27]. We used United States Dollars (USD) for all currency measurements. Table 1 shows the variables and sources used for those items.

### 2.3. Operationalization of Study Variables

Consistent with other studies, the dependent variable we used to assess the organization’s overall financial performance was return on assets, or ROA [1,2]. ROA has been consistently used in financial literature and is calculated as follows:

Return on Assets (ROA) = (Net Income/Total Assets) × 100

A higher ROA is indicative of increased market performance over competing hospitals and indicates greater profitability from assets invested in the organization. ROA also reflects the ability of a hospital to meet its existing operational needs as well as its capability to generate capital for any future needs [28].

Several moderating and covariate variables were also examined to better understand a hospital’s financial stability, including net patient care reimbursements per adjusted patient day, which account for the amount collected per unit of service for a hospital. We also included non-operating income, such as auxiliary income from parking, cafeterias, and bookstores, research grants, donations, and, most importantly, financial investment income. Unique to the years examined in this study, COVID-19 assistance from government funding like The Coronavirus Aid, Relief and Economic Securities Act (The CARES Act) and the Paycheck Protection and Health Care Enhancement Act may have had an impact on non-operating income [29]. Teaching hospitals, in particular, received a greater portion of available funding from these sources when compared to non-teaching hospitals [16]. While private facilities without a teaching mission may rely less on that non-operating revenue, larger organizations with a teaching element have a greater reliance on non-operating revenue streams [30].

In order to further evaluate the impact of CARES Act funding on hospitals in the study population, we obtained payment data by hospital under the CARES Act through 2022 [31]. The payment data we obtained from HRSA included total payments through 2022 and did not segregate payments by provider among the four phases of funding disbursed during 2020, 2021, or 2022. However, total disbursements for each of the phases were published, and those totals were used to prorate the total payment by hospital among the years in this study, and we applied those payments in 2020 and 2021 in our study. Even though CARES payment data for 2022 was available, HCRIS data for 2022 was not available for all facilities, so the assessment of CARES Act payments was limited to the years 2020–2021 in our study. Of all CARES Act payments, 80% of those funds were disbursed in 2020 and 2021, with 65% of those payments occurring in 2020 [31].

Our analysis seems to support the notion that non-operating funds were significant in boosting ROA during the years 2020 and 2021, as the facilities in this study on average incurred a loss from patient care services yet earned an overall net income due to the extent of non-operating revenues earned. Of that non-operating income increase between 2019 and the pandemic years of 2020 and 2021, CARES Act funding averaged. This data is summarized in Table 2.

Total cash available to a facility indicates financial strength and the ability to continue paying expenses. We measured cash based on days of cash on hand, which was calculated by total cash divided by average daily cash expenses [20]. In this analysis, we considered depreciation as the primary non-cash expense and deducted the amount of depreciation from total operating expenses to estimate cash expenses to the organization for each year [32]. No other non-cash items, such as amortization of long-term items, are segregated in the Medicare cost report and so are not delineated in our analysis. This may represent a limitation to our work to the extent that there is an unrecognized amortization in a hospital’s annual expenses that is not treated as a non-cash expense. To the extent that any such item exists in our study, it would have a potential bias that would reduce our estimate of days of cash on hand.

### 2.4. Moderating and Control Variables

We used the year as the primary moderating variable. It was expected that the year 2020 would be significantly impacted by COVID-19. We collected data for complete fiscal years, including 2019, 2020, and 2021. We also used geographic regions, coded based on the U.S. Census Bureau’s classification of the region for each hospital [33]. Region 1 is the Northeast; region 2 is the Midwest; region 3 is the South; and region 4 is the West. Hospitals at higher risk for financial challenges have been identified empirically as those in small rural communities [4]. Controlling for region may reduce the impact of region on operating margin. Many studies imply geography is critical to the financial health of a hospital, with rural hospitals being at greater risk for financial instability than urban hospitals [4,14,34,35]. Owing to the fact that all hospitals identified in this sample were located in urban areas, we did not control for this variable in our analysis.

### 2.5. Statistical Analysis

Both the region of hospital location and the fiscal year of data collection were treated as categorical variables. We conducted multivariate linear regression analysis to determine the association between the independent variables and each study outcome and reported coefficients, betas and significances. Next, we constructed three multivariable linear regression analysis models, one for each study outcome. The main effects were not included in the interactions of the fiscal year with the covariates. Margin analyses were conducted, and graphs were examined for the main effect covariates as well as the significant interactions to determine if fiscal year was a moderator of the association between a covariate and the study outcome being analyzed. We reported coefficients and betas for covariates in the models and figures illustrating the marginal effects of the fiscal year. A probability value of <0.05 (two tails) was considered statistically significant for all tests. We performed all statistical analyses using Stata IC 17 [36].

## 3. Results

The median ROA for all hospitals in this study ranged from 6% in 2019, slipping to 4% in 2020, and rebounding to 8% in 2021. During that same time frame, there was a significant decrease in income from patient services from 2019 to 2021, suggesting that the revenues from patient care were not sufficient to cover the costs of delivering care during the time of high COVID-19 incidence. This income measure rebounded in 2021 but did not reach levels seen in 2019 before the pandemic. Operating losses were approximately 4.7%, down from a 9.8% loss in 2020 and a 1.2% loss in 2019. However, non-operating income increased significantly from 2019 to 2020, with a decline in 2021, but levels were still above the 2019 baseline in our data. As mentioned earlier, CARES Act funding was a major contributor to that observation, with the majority of the year-over-year increase in non-operating income (averaging 75%) from 2019 to 2020 coming from this source. An average of 22.5% of the increase from 2019 to 2021 came from CARES Act funding. The remainder of the increase can be attributed to increases in investment income [37].

Within the observed losses from patient care services, net revenues per adjusted occupied bed increased an average of 6.8% from 2019 to 2020, which appeared in line with the 7.0% increase in case-mix index across the same timeframe. Going from 2020 to 2021, net revenues remained relatively flat, increasing only 0.5%, as did the average case mix index. Thus, reimbursement appeared to correlate with the relative severity of the illness being treated. However, the costs of treating patients increased faster than revenues from 2019 to 2020, with an average annual increase of 14%. Salaries and other operating expenses increased in nearly equal proportions. This observation makes sense given the widespread difficulties with obtaining the staff needed to meet patient care needs and the widespread supply chain problems that drove up the costs of medications and personal protective equipment in 2020. Staffing challenges required hospitals to contract traveling nurse staff at rates that were significantly higher.

From a cash flow perspective, it was interesting to note that days in accounts receivable increased only slightly in regions 1, 2, and 4 from 2019 to 2020 and also from 2020 to 2021, while declining in region 3 in both years. This observation was caused by one facility with unusually high days in receivables in 2019 that brought this ratio to the regional average by the end of 2021. When removing this one large observation from the calculation, the rest of the hospitals in the region showed slight increases, as was noted in the other three regions in this study.

The overall increase in accounts receivable, as well as cash on hand increases arising from increased non-operating revenue collections in 2020, had the effect of increasing total assets in all four regions in 2020. This increase in assets, coupled with declines in net income during the same time, led to a decline in return on assets in regions 1, 2, and 4. Region 3 experienced an increase in ROA from 2019 to 2020, arising from an increase in net income. That increased net income in region 3 was driven by two facilities having large increases in non-operating revenue during both 2020 and 2021. Controlling for those two facilities, the remainder of the region also experienced a decline in ROA during both years when compared with 2020.

We then prepared a multivariate robust regression analysis to control for potential serial correlation among variables, using ROA as the dependent variable. Owing to differences in scale of operation and service mix, we attempted to control for these factors by using revenue, salary, and operating expense measures on a per-adjusted patient-day basis. Changes in payer mix and uninsured patient care responsibilities, along with service mix and differences in reimbursement levels across a nationwide sample, could influence usage of labor resources and so could impact measures for staffing (FTE per adjusted occupied bed), with non-operating income, accounts receivable/total asset ratio, debt ratio, region, and fiscal year included in the final model. We found that overall, lower cash holdings, higher non-operating income, and lower accounts receivable balances had the most significant relationships with ROA. Considering that the net income increased across the study period while the asset base used in that ratio remained relatively stable, the observation makes sense—considering the impact of non-operating income on net income in the study hospitals. Table 3 presents a summary of our regression model, listing those variables with significant results (*p* < 0.05). A full listing of results for all variables in the regression model is listed in Appendix A.

**Figure 1:**
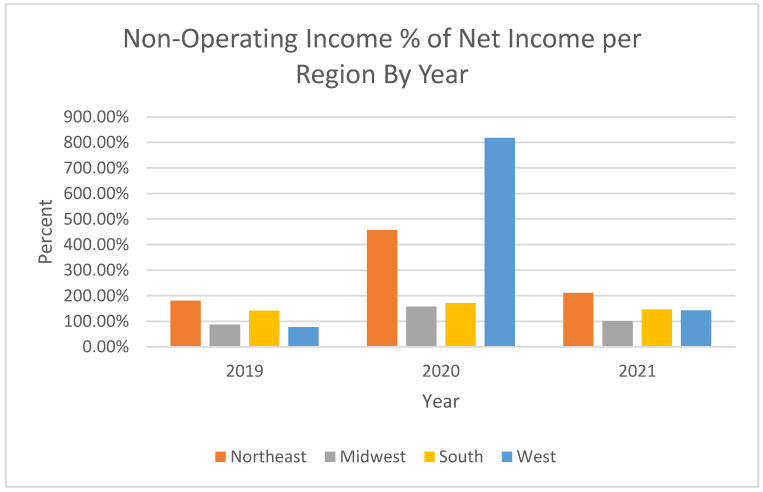
Non-Operating Income as a percentage of Net Income, by Region.

Interesting in the review of regression results in Table 3 is that the dummy variables representing region were not statistically significant for all years in this study, suggesting that the observed changes in ROA appeared to be a nationwide outcome. The dummy variable for the year 2020 was not statistically significant. However, the dummy variable for 2021 was statistically significant, suggesting that the overall change in ROA from 2020 to 2021 was significant. Revenue and expense measures per adjusted patient day were significant, as would be expected with those elements being the components of the net income used to derive ROA. Most important in evaluating these observations was that the variable for non-operating income as a percentage of net income was statistically significant (*p* = 0.003), indicating that this variable appeared to be an important driver of the observed changes in ROA across the study period. Figure 1 below illustrates each region’s non-operating Income as a percentage of net income across the study time periods.

## 4. Discussion

This research focuses on the impact that the COVID-19 pandemic had on U.S. teaching hospitals for the years 2019–2021. Our study found evidence that patient income was significantly reduced, yet margins increased in the latest year largely due to additional funding from non-operating incomes. The Coronavirus Aid, Relief and Economic Securities Act (The CARES Act) appropriated nearly $170 billion for medical care of COVID-19 cases, 60% of which was earmarked for healthcare provider reimbursement for lost revenues [23] and seems to have had an impact during this period. Considering the decline in income from patient care services in 2020, the impact of non-operating income, such as the CARES Act funding, was a significant factor in the generation of overall net incomes across the years 2020 and 2021.

Patient revenues were higher during the height of the pandemic in 2020 and into 2021. However, higher operating expenses (a factor of higher salaries and the costs of additional personal protective equipment) and greater staffing needs within the ICU (due to an unprecedented amount of widespread use of ventilator care during the initial wave of COVID-19 hospitalizations) offset any patient care income gains arising from increased patient volumes, as hospitals had to utilize various strategies to address workforce challenges during the pandemic. To reiterate, the effect of non-operating income on the bottom line of this set of hospitals cannot be diminished, considering the extent of losses incurred from patient care during the study period.

These findings generally align with those of Cantor et al., who noted that hospitals may have benefited from the CARES Act funds, with funds disproportionately going to hospitals that had higher assets pre-COVID [16]. Since CARES Act funds represent contributions or non-operating income, these receipts increased that element of the income statement observed in our study [38]. Our results demonstrate the positive impact of non-operating income on ROA during the 2019 to 2021 period.

### 4.1. Strengths and Limitations

One of the strengths and uniqueness of this study is the analysis of major teaching hospitals by revenue in the United States, along with a comparison of whether key measures of financial performance have improved during the most recent fiscal year, post-COVID-19. This study provides an observational, cross-sectional analysis of how hospitals fared during the pandemic and its immediate aftermath.

This study is not without limitations. As with all secondary analyses, the financial and operating data are lagged. Ideally, we would focus on the end of FY2022 data to better understand how facilities are performing, but these data are not yet available. In addition, we did not attempt to focus on all variables impacting hospital strategy but rather focused on the core financial and operating variables to better understand how teaching hospitals are performing amidst this novel pandemic. Future research should closely examine the best practices in operating efficiency that some facilities are able to achieve. Implementing Lean, Six Sigma, and other process improvement techniques today could offer one potential strategy [39].

### 4.2. Areas of Future Research

This research study can be used as a foundation for multiple future research studies. One area of future research can be the expansion of this study’s design and its extension to all teaching hospitals in the U.S. A second area of future research can be to expand this study’s design to all hospitals, not just the teaching hospitals, in the U.S. and globally. A third area of future research can explore organizational management theories, like transaction cost economics, resource dependence theory and institutional theory, and operationalize them to study the financial performance of hospitals in the post-pandemic environment.

Quantitative studies are not the only options for future research. This study’s findings can also be utilized for qualitative studies that look for themes that may emerge from observations and evaluations of certain contexts. Semi-structured or structured interviews with various decision-makers and members of the leadership team at teaching hospitals can be conducted to ascertain a more holistic understanding of the strategies they have used and are using to navigate the challenges faced by their hospitals. Additionally, mixed-methods studies can also be conducted based on this study’s findings, incorporating both quantitative and qualitative research methodologies.

## 5. Conclusions

In this study of 104 of the largest teaching hospitals in the U.S., our analyses suggest that financial positions improved in 2021 for both ROA and operating margins after a downturn during the height of the COVID-19 pandemic in 2020. However, material increases in operating expenses pose a threat to the financial viability of these important healthcare organizations. Changes in operating expenses outpaced operating revenues during 2020 and 2021. It, therefore, seems incumbent on managers in teaching hospitals to seek out ways to expand and maintain non-operating revenues to offset losses from patient care services. These findings suggest an important need for initiatives encouraging efficiency and operating expense control, especially in the fixed-cost administrative departments of the hospital. As revenues rise or fall with patient volumes, these administrative costs remain static. During a pandemic event where service volumes and revenues could be adversely impacted, the ability to pay those costs not directly related to service volumes could reduce ROA if non-operating subsidies such as CARES Act funding are not available in a future pandemic event [40,41]. We recommend a strategic focus on building up financial reserves today, building out non-operational revenue streams, and implementing operational efficiencies to foster better financial resiliency, which might enable facilities to adapt better to future pandemics and environmental turbulence.

## Figures and Tables

**Table 1:** Variables and Data Sources.

Variable	Source
Return on Assets	Net income/Total Assets
Cash and Temp Investments	HCRIS, Worksheet G, Lines 1 and 2, Columns 1–4
Accounts Receivable	HCRIS, Worksheet G, Line 4, Columns 1–4
All Other Assets	Total Assets—Cash and Temp Investments—Accounts Receivable
Total Assets	HCRIS, Worksheet G, Line 36, Columns 1–4
Cash and Temp Investments % of Assets	(Cash and Temp Investments/Total Assets) × 100
Accounts Receivable % of Assets	(Accounts Receivable/Total Assets) × 100
Total Liabilities	HCRIS, Worksheet G, Line 51, Columns 1–4
Net Revenues	HCRIS, Worksheet G-3, Line 3, Column 1
Total Salary Expense	HCRIS, Worksheet A, Line 200, Column 1
Non-Salary Operating Expense	Total Operating Expense—Total Salary Expense
Total Operating Expense	HCRIS, Worksheet G-3, Line 4, Column 1
Income from Patient Services	HCRIS, Worksheet G-3, Line 5, Column 1
Non-Operating Income	HCRIS, Worksheet G-3, Line 25, Column 1 HCRIS, Worksheet G-3, Line 28, Column 1
Net Income	HCRIS, Worksheet G-3, Line 29, Column 1
Days in Accounts Receivable	Accounts Receivable/(Net Revenues/365)
Days Cash on Hand	Cash and Temp Investments/[(Total Operating Expense—HCRIS, Worksheet A-7, Part III, Line 3, Column 9)/365]
FTE Employees per Adjusted Occupied Bed	(HCRIS, Worksheet S-3, Part 3, Line 1, Column 5/2080)/[(HCRIS, Worksheet S-3, Part I, Line 14, Column 8/365) × (HCRIS, Worksheet G-2, Line 28, Column 3/HCRIS, Worksheet G-2, Line 28, Column 1)]
Net Revenue per Adjusted Occupied Bed	Net Revenue/[(HCRIS, Worksheet S-3, Part I, Line 14, Column 8/365) × (HCRIS, Worksheet G-2, Line 28, Column 3/HCRIS, Worksheet G-2, Line 28, Column 1)]
Operating Expense per Adjusted Occupied Bed	Total Operating Expense/[(HCRIS, Worksheet S-3, Part I, Line 14, Column 8/365) × (HCRIS, Worksheet G-2, Line 28, Column 3/HCRIS, Worksheet G-2, Line 28, Column 1)]
Salaries as a % of Net Revenue	(Total Salary Expense/Net Revenue) × 100
Non-Operating Income % of Net Income	(Non-Operating Income/Net Income) × 100
Case Mix Index—*measure of relative severity of illness treated*	CMS Case Mix Index Files from Hospital Inpatient PPS reimbursement files at https://www.cms.gov/Medicare/Medicare-Fee-for-Service-Payment/AcuteInpatientPPS (accessed on 12 December 2022)

**Table 2:** Means by Year and Region for Study Variables.

	Region 1			Region 2			Region 3			Region 4		
Variable	2019	2020	2021	2019	2020	2021	2019	2020	2021	2019	2020	2021
Return on Assets	0.070	0.036	0.058	0.122	0.103	0.165	0.062	0.074	0.091	0.059	0.016	0.067
Cash and Temp Investments ($,000)	346,402	547,859	468,336	295,262	369,238	363,931	279,397	414,269	391,247	434,269	643,425	602,685
Accounts Receivable ($,000)	299,024	303,874	379,971	518,210	483,271	638,938	486,650	452,812	522,868	571,976	591,711	778,152
All Other Assets ($,000)	1,059,522	1,189,465	1,528,332	1,732,226	1,964,358	2,094,191	1,399,877	1,771,350	2,359,178	1,880,774	2,168,394	2,183,128
Total Assets ($,000)	1,704,948	2,041,199	2,376,638	2,545,697	2,816,866	3,097,059	2,165,924	2,638,432	3,273,293	2,887,019	3,403,530	3,563,965
Total Liabilities ($,000)	922,036	2,041,199	1,296,356	943,313	1,197,479	1,228,021	856,105	1,239,568	1,607,560	1,897,319	2,351,859	2,306,883
Net Revenues ($,000)	1,516,811	1,477,582	1,704,113	1,618,305	1,580,457	1,766,109	1,534,341	1,602,633	1,793,934	1,890,459	1,940,560	2,049,296
Total Salary Expense ($,000)	508,802	546,379	561,116	486,257	499,358	518,733	510,149	546,536	634,490	610,346	677,109	715,952
Non-Salary Operating Expense ($,000)	1,098,769	1,183,189	1,304,089	1,112,915	1,154,404	1,248,957	1,080,747	1,172,549	1,274,944	1,241,580	1,417,961	1,439,628
Total Operating Expense ($,000)	1,607,572	1,729,568	1,865,205	1,599,172	1,653,762	1,767,690	1,590,896	1,719,085	1,909,433	1,851,925	2,095,070	2,155,580
Income from Patient Services ($,000)	(90,672)	(251,986)	(161,092)	19,133	(73,305)	(1581)	(56,555)	(116,452)	(115,499)	38,534	(154,510)	(106,284)
Non-Operating Income ($,000)	202,930	322,543	306,318	130,773	201,632	272,821	194,139	280,764	366,012	127,173	176,036	356,121
Estimated CARES Act Funding ($,000)	0	83,382	44,897	0	47,729	25,701	0	53,329	28,662	0	49,591	26,703
Net Income ($,000)	112,169	70,556	145,226	149,906	128,327	271,240	137,583	164,313	250,513	165,707	21,525	249,837
Days in Accounts Receivable	73.32	75.34	83.08	128.36	129.59	146.05	132.80	120.12	118.11	131.59	132.00	180.11
Days Cash on Hand	64.39	100.39	79.60	78.17	100.55	95.73	67.60	97.42	85.98	71.58	97.20	90.73
FTE per Adjusted Occupied Bed	6.43	7.41	6.98	6.14	6.39	6.06	6.19	6.45	6.05	7.86	9.51	8.30
Net Revenue/Adjusted Occupied Bed	4073.49	4226.18	4345.16	3932.94	4091.52	4241.01	3415.50	3833.50	3868.81	6418.08	6881.83	6521.85
Operating Expense per Adjusted Occupied Bed	4312.93	4985.01	4785.31	3901.89	4295.98	4261.24	3528.39	3996.84	4118.82	6341.46	7448.87	6892.99
Salaries as a % of Net Revenue	33.54%	36.98%	32.93%	30.36%	31.79%	29.78%	33.93%	35.95%	39.81%	32.32%	34.07%	34.21%
Non-Operating Income % Net Income	180.91%	457.14%	210.93%	87.24%	157.12%	100.58%	141.11%	170.87%	146.11%	76.75%	817.80%	142.54%
Case Mix Index	2.036	2.176	2.178	2.171	2.323	2.326	2.151	2.332	2.333	2.362	2.500	2.503
Total Facilities	24			28			33			19		

**Table 3:** Multivariate Regression Variables with Significant Results.

ROA	Coefficient	Robust Std. Err.	*p* > |t|
Net revenue per adjusted occupied bed	0.00010	0.00002	0.000
Salary expense per adjusted occupied bed	−0.00017	0.00005	0.001
Non-salary expense per adjusted occupied bed	−0.00006	0.00001	0.000
Non-Operating Income % of Net Income	0.00009	0.00003	0.003
Days in Accounts Receivable	−0.00017	0.00007	0.017
Accounts Receivable % of Assets	0.10648	0.02957	0.000
Dummy variable 2021	0.03631	0.01124	0.001

## Data Availability

This study used publicly available secondary data, and the data sources have been provided in Table 1.

## Appendix A. Full Regression Results

**Variable**	**Coefficient**	**Robust Std. Err.**	**P > |t|**
Net revenue per adjusted occupied bed	0.00010	0.00002	0.000
Salary expense per adjusted occupied bed	−0.00017	0.00005	0.001
Non-salary expense per adjusted occupied bed	−0.00006	0.00001	0.000
Salary expense as a % of net revenue	26.23699	13.56722	0.054
FTE employees per Occupied Bed	0.00357	0.00404	0.378
Non-Operating Income % of Net Income	0.00009	0.00003	0.003
Days Cash on Hand	−0.00004	0.00009	0.642
Days in Accounts Receivable	−0.00017	0.00007	0.017
Accounts Receivable % of Assets	0.10648	0.02957	0.000
Cash and Temp Investments % of Assets	0.06326	0.05442	0.246
Dummy variable 2020 (base = 2019)	0.01220	0.01143	0.287
Dummy variable 2021	0.03631	0.01124	0.001
Case-Mix Index	−0.02913	0.02141	0.175
Constant	−0.01454	0.05778	0.802
